# Prognosis for patients diagnosed with pregnancy-associated breast cancer: a paired case-control study

**DOI:** 10.1590/S1516-31802010000300003

**Published:** 2010-05-06

**Authors:** Wagner Brant Moreira, Eduardo Carvalho Brandão, Aleida Nazareth Soares, Clécio Enio Murta de Lucena, Carlos Maurício Figueiredo Antunes

**Affiliations:** I MD, MSc. Attending physician at the Clinical Oncology Service, Hospital da Santa Casa de Misericórdia and medical consultant at Centro de Quimioterapia Antiblástica e Imunoterapia (CQAI), Belo Horizonte, Minas Gerais, Brazil.; II MD. Attending physician at the Clinical Oncology Service, Hospital da Santa Casa de Misericórdia, and at Centro de Quimioterapia Antiblástica e Imunoterapia (CQAI), Belo Horizonte, Minas Gerais, Brazil.; III MSc. Biostatistician at the Clinical Oncology Service, Hospital da Santa Casa de Misericórdia, and at Centro de Quimioterapia Antiblástica e Imunoterapia (CQAI), Belo Horizonte, Minas Gerais, Brazil.; IV PhD. Attending physician at the Mastology Service, Hospital da Santa Casa de Misericórdia, Belo Horizonte, Minas Gerais, Brazil.; V PhD. Professor, Parasitology Department, Universidade Federal de Minas Gerais (UFMG), and member of the Postgraduate Studies Board of the Clinical Medicine and Biomedicine Department, Hospital da Santa Casa de Misericórdia, Belo Horizonte, Minas Gerais, Brazil.

**Keywords:** Breast neoplasms, Prognosis, Pregnancy, Lactation, Pregnancy complications, Neoplasias da mama, Prognóstico, Gravidez, Lactação, Complicações na gravidez

## Abstract

**CONTEXT AND OBJECTIVE:**

Previous studies have suggested that the occurrence of pregnancy concomitantly with a diagnosis of breast cancer may affect the evolution of the neoplasia. The present study aimed to compare pregnancy-associated breast cancer (PABC) patients with non-pregnant cancer patients (controls) in relation to the time taken to diagnose the disease, tumor characteristics and mortality.

**DESIGN AND SETTING:**

A retrospective, paired case-control study was conducted at the Hospital da Santa Casa de Misericórdia and Centro de Quimioterapia Antiblástica e Imunoterapia in Belo Horizonte, Brazil.

**METHODS:**

The study involved 87 PABC and 252 control patients. The influence of covariables (interval between first symptoms and diagnosis, tumor histology, size of primary tumor, distant metastasis, grade of malignancy, hormone receptor status and axillary lymph node involvement) and the pregnancy variable on overall survival was investigated using univariate and multivariate analyses.

**RESULTS:**

The median overall survival for PABC patients of 30.1 months (95% confidence interval, CI: 19.4-40.9 months) was significantly different (P = 0.005) from that of the control group (53.1 months; 95% CI: 35.1-71.0 months). The cumulative overall survivals after five and ten years were, respectively, 29.7 and 19.2% for PABC patients, and 47.3 and 34.8% for control patients (P = 0.005). Tumor size, grade of malignancy, distant metastasis and pregnancy were independent factors that significantly modified disease prognosis.

**CONCLUSIONS:**

Pregnancy was an independent prognostic factor. The overall survival of PABC patients was shorter than that of non-pregnant patients.

## RESUMO

**CONTEXTO E OBJETIVO:**

Estudos prévios sugerem que a ocorrência de gravidez simultaneamente com o diagnóstico de câncer de mama pode afetar a evolução da neoplasia. O presente estudo objetivou comparar pacientes com câncer de mama associado à gravidez (PABC) e pacientes com câncer não grávidas (controles) com relação ao tempo decorrido até o diagnóstico de câncer, características tumorais e mortalidade.

**TIPO DE ESTUDO E LOCAL:**

Estudo retrospectivo, tipo caso-controle pareado, foi conduzido no Hospital da Santa Casa de Misericórdia e Centro de Quimioterapia Antiblástica e Imunoterapia em Belo Horizonte, Brasil.

**MÉTODOS:**

O estudo envolveu 87 pacientes PABC e 252 controles. A influência das covariáveis (intervalo entre os primeiros sintomas e diagnóstico, histologia do tumor, tamanho do tumor primário, metástase a distância, grau de malignidade, dosagem de receptor hormonal e acometimento dos linfonodos axilares) e da variável gravidez sobre a sobrevida global foram avaliados através de análises univariada e multivariada.

**RESULTADOS:**

A mediana da sobrevida global para as pacientes PABC de 30,1 meses (intervalo de confiança, IC 95%: 19,4-40,9 meses) foi significativamente (P = 0,005) diferente daquela dos controles (53,1 meses; IC 95%: 35,1-71,0 meses). A sobrevida global acumulada após 5 e 10 anos foi 29,7 e 19,2% no grupo PABC e 47,3 e 34,8% nos controles. Tamanho do tumor, grau de malignidade, metástase a distância e gravidez foram fatores independentes e significativamente condicionantes do prognóstico da doença.

**CONCLUSÕES:**

A gravidez foi um fator independente para o prognóstico da doença. A sobrevida global das pacientes PABC foi mais curta do que aquela dos controles.

## INTRODUCTION

Conventionally, breast cancer is classified as pregnancy-associated breast cancer (PABC) when the disease is diagnosed during pregnancy or up to one year after parturition.^1^ In the United States, breast cancer is diagnosed in one in every 3,000 to 10,000 pregnant women, resulting in some 3,500 new cases annually.^1^ In Canada, the incidence of PABC is somewhat higher (1 case in 1,500 to 4,000 pregnant women).^2^ According to DiFronzo and O'Connell,^3^ breast cancer affects between 0.2 and 3.8% of all pregnant women, an incidence that is apparently increasing as a result of delayed pregnancy (later than 30 years of age)^2^ and higher levels of detection (by means of mammography) of breast cancer in younger women.^1,4^

One of the earliest suggestions of an association between pregnancy and unfavorable evolution of breast cancer was published by White in 1954, who noted that less than 20% of pregnant women who developed carcinoma of the breast survived for more than five years following diagnosis.^5^ A number of subsequent studies have provided evidence supporting this pessimistic prognosis for PABC patients. Ishida et al.^6^ concluded that delayed diagnosis and treatment, together with a more aggressive condition, were responsible for the poor prognosis among pregnant or lactating women suffering from breast cancer. The deleterious effect of pregnancy on the prognosis of women diagnosed with neoplasia has been confirmed even when axillary lymph node involvement and treatment of the primary tumor are taken into consideration.^7^ Additionally, multivariate analysis carried out on data obtained from PABC patients in a multicenter study has revealed that when cancer is diagnosed during pregnancy or the postpartum period, the prognosis for the patient is pessimistic.^8^ A recent population-based study involving a large number of patients has substantiated the negative prognosis for women suffering from PABC.^9^

In contrast to the above, several studies have been unable to confirm any association between prognosis and pregnancy in PABC patients. Petrek et al.^10^ investigated axillary lymph node involvement and pregnancy as possible predictive factors for the development of breast cancer in pregnant and non-pregnant women of the same age range, each in various stages of the disease. Only axillary lymph node involvement was decisive for the prognosis of the disease outcome and, when the stage of the illness was taken into consideration, there were no significant differences between the two groups. Similarly, no differences were found with regard to the median and overall survival rates between a group of pregnant women diagnosed with breast cancer and non-pregnant women in the same condition.^11^ Considering the relevance of the subject and the lack of information relating to Brazilian patients suffering from PABC, we undertook a retrospective study from which the results are reported in this paper.

## OBJECTIVE

The objective of this study was to compare pregnancy-associated breast cancer (PABC) patients with non-pregnant patients presenting breast cancer (controls), in relation to the time taken to diagnose the disease, the tumor characteristics and mortality.

## METHODS

### Type of study

This paired case-control study consisted of a retrospective, longitudinal analysis on two groups of women suffering from breast cancer. The PABC group consisted of patients diagnosed either during pregnancy or up to 12 months after parturition or abortion, while the control group consisted of women with similar characteristics but with no recent history (within 12 months) of pregnancy or parturition at the time when the symptoms emerged or when the diagnosis was reached.

### Setting

The study covered the period between January 1980 and December 2000 at the Immunotherapy and Antiblastic Chemotherapy Center (Centro de Quimioterapia Antiblástica e Imunoterapia; CQAI) and the Clinical Oncology Service, Hospital da Santa Casa de Misericórdia, in Belo Horizonte, Minas Gerais, Brazil. Details of the project were presented to, and approved by, the Ethics Committee of Hospital da Santa Casa de Misericórdia prior to commencing the study.

### Sample

Data on 1,521 women (≤ 45 years old) diagnosed with breast cancer at the Clinical Oncology Service were obtained from medical records kept at the Oncological Research and Study Center of Minas Gerais (Centro de Estudos e Pesquisas Oncológicas de Minas Gerais; CEOMG). The PABC (n = 87) and control (n = 252) patients were classified according to the institution at which the patient had been registered, the date of registration and the individual's age. Each PABC patient was matched with corresponding control patients in accordance with the following criteria: (i) registration in the same institution, (ii) similar age (within two years), and (iii) similar registration year (within two years). For cases in which more than three control patients matched a single PABC patient, selection was based on the closest registration date.

Following identification of the total PABC population (n = 93), an overall sample size was calculated assuming that all study variables contained a maximum of three categories (≤ 2 degrees of freedom) and that the objective was to measure a mean effect to be detected statistically, with a significance level of 5% and a power of detection ≥ 80%. The calculations indicated that the total number of patients should be within the range of 200 to 250, according to Cohen's effect size conventions.^12^ In practice, a larger number of controls was selected (three for each PABC patient) as a precaution against any need to exclude some of the individuals. Thus, 372 patients were available for the study. However, the final population selected (n = 339) ensured that the power of the statistical tests for detecting a mean effect (at the 5% significance level) was approximately 90%.^12^

### Main measurements

The covariables identified from the patients’ medical records that could potentially be associated with disease prognosis included grade of malignancy, tumor histology, size of the primary tumor, axillary lymph node involvement, presence of distant metastasis, hormone (estrogen or progesterone) receptor status, interval between first symptoms and diagnosing of cancer, and interval between diagnosis and first treatment. The variable used to assess the prognosis was the overall length of survival, which was defined as the interval between diagnosing the breast cancer and the patient's death (or the date of the last information obtained).

### Statistical analysis

The overall survival functions of the two groups of patients were analyzed in accordance with the Kaplan-Meier method and compared using the Mantel-Cox log rank method. The evaluation of covariables followed the methodology described by George,^13^ and was based on univariate analysis that aimed to identify the covariables that were individually related to the prognosis. Covariables were considered to be significantly associated with the prognosis when the differences between the two groups presented a P value ≤ 0.20 (χ^2^ test). Potentially important factors were included in a Cox multivariate proportional hazard regression model that was constructed with the objective of identifying the covariables that independently influenced the prognosis. Statistical analyses were performed using Epi-Info (version 3.3.2; February 2005) and the Statistical Package for the Social Sciences (SPSS) software, version 13.0.

## RESULTS

### Patients’ characteristics

Out of the total of 1,521 women (≤ 45 years old) suffering from breast cancer, 93 PABC patients (6.1%) were identified. On the basis of matching criteria, 252 women were selected as controls. Of the PABC patients, six (6.45%) could not be matched with any of the controls (and thus were omitted from the study), one was matched with a single control, seven were matched with two controls each, whilst the remainder (79 women) were matched with three controls each.

Table 1 presents the distribution of patients according to the covariables selected as having potential associations with disease prognosis. Data on the covariables were available for more than 90% of the PABC and control individuals, with the exception of those relating to grade of malignancy and hormone receptor status (Table 1). Lack of information on any of the covariables can negatively affect the quality of a prognosis study,^13^ although it is generally agreed that a maximum of 20% of cases with incomplete data is acceptable. In the present study, however, more than 20% of cases were deficient with regard to data relating to the grade of malignancy and hormone receptor status. Since there were no significant differences (as indicated by P values > 0.05) between the PABC and control groups (either including or excluding the cases with missing data) in relation to the grade of malignancy and hormone receptor status, we chose to include these two covariables in the statistical analysis and to examine the cases with incomplete data separately.

**Table 1.** Characteristics of pregnancy-associated breast cancer (PABC) and control breast cancer patients (not pregnant)

Characteristics	PABC group (n = 87)*	Control group (n = 252)	P
Age (years)			
Median value (25^th^ - 75^th^ percentile)	35.0 (31 - 39)	36.0 (32 - 39)	
Mean value ± standard error	34.9 ± 4.89	35.5 ± 4.83	
Tumor histology (% of cases)			
Ductal/lobular	79 (90.8)	232 (92.1)	0.713†
Other	8 (9.2)	20 (7.9)	
Grade of malignancy (% of cases)			
1 and 2	20 (23.0)	61 (24.2)	0.375†
3	22 (25.3)	81 (32.1)	
Unknown	45 (51.7)	110 (43.7	
Primary tumor size (% of cases)			
T_1_ + T_2_	25 (28.7)	97 (38.8)	0.029†
T_3_ + T_4_	61 (70.1)	138 (55.4)	
Unknown	1 (1.10)	14 (5.80)	
Axillary lymph node involvement (% of cases)			
Negative	9 (10.3)	161 (63.9)	< 0.0001†
Positive	78 (89.7)	91 (36.1)	
Distant metastasis (% of cases)			
Negative	57 (65.5)	202 (80.2)	0.007†
Positive	29 (33.3)	49 (19.4)	
Hormone receptor status (% of cases)			
Negative	12 (13.8)	39 (15.5)	0.226†
Positive	39 (44.8)	87 (34.5)	
Not determined	36 (41.4)	126 (50.0)	
Interval between first symptoms and diagnosis (% of cases)			
≤ 6 months	32 (36.8)	129 (51.2)	0.009†
> 6 months	55 (63.2)	114 (45.2)	
Interval between diagnosis and first treatment (days)			
Mean value ± standard error	79.20 ± 377	51.61 ± 109	0.402‡
Median value	22.0	28.5	

* Excludes six PABC patients with no matching controls;

† Values based on χ^2^ test;

‡ Value based on Mann-Whitney test. Staging and grade of malignancy according to the TNM system.

The PABC group presented significant differences in comparison with the control group, in relation to primary tumor size, axillary lymph node involvement, presence of distant metastasis and duration of symptoms before diagnosis. Information was unavailable concerning disease outcome for 26.3% (89/339) of the remaining individuals involved in the study, including 24.1% (21/87) of the PABC group and 26.9% (68/252) of the control group. Nine PABC patients and 18 control patients entered the terminal phase during hospitalization and were discharged at the request of their families, a practice that was common in the 1980s and the early part of the 1990s. Attempts to obtain information concerning the fate of these patients generated anxiety among family members, and hence we considered that these patients died on the date of the last information registered. Thus, it may be assumed that 73.6% (64/87) of the PABC group and 59.1% (149/252) of the control group died as a consequence of breast cancer: the difference in these values was statistically significant, as indicated by the χ^2^ test (P < 0.05).

Among the remaining 62 patients (18.2%; 62/339) whose outcomes were unknown, 21 were followed up for a period ≥ 60 months (including eight who were followed up for ≥ 120 months). On this basis, information on 41 patients (12.1%) who were followed up for a period of < 60 months was considered to be missing data. This situation imposed some difficulties on prognosis analysis and, in order to overcome the problem, patients discharged from hospital whose outcomes were unknown were classified as “missing/gravely ill”. The data on these patients were analyzed assuming that this classification represented either death or censored data (i.e. losses from the sample before the final outcome was observed).

### Overall survival

The Kaplan-Meier survival estimator function was applied to data derived from all of the 339 patients studied, and the data in the missing/gravely ill category were considered either to represent cases of death or censored data. The results obtained are summarized in Table 2. Classification of missing/gravely ill data as being equivalent to an outcome of death resulted in a worse prognosis than when such data were classified as censored. However, irrespective of the classification used, the difference in overall survival between the PABC and control groups was highly significant, as shown by the P value of 0.005 calculated according to the Mantel-Cox log rank method. It is likely that values intermediate between the two sets of results shown in Table 2 would have been obtained from the analysis, had all data been available. In order to simplify the treatment of the results, we chose to classify missing/gravely ill data as equivalent to death in subsequent analyses.

**Table 2.** Overall survival of pregnancy-associated breast cancer (PABC) and control breast cancer patients

Treatment data	Overall survival				
	Median (months)	95% CI*	5 years (%)	10 years (%)	P†
a) Missing/gravely ill data equated to *death*					
Control group	53.1	35.1 - 71.0	47.3	34.8	0.005
PABC group	30.1	19.4 - 40.9	29.7	19.2	
b) Missing/gravely ill data equated to *censored*					
Control group	70.1	50.5 - 89.4	52.3	40.3	0.004
PABC group	32.6	21.8 - 43.5	32.8	21.2	

* 95% confidence interval;

† Determined in accordance with Mantel-Cox log rank method.

**Figure 1:**
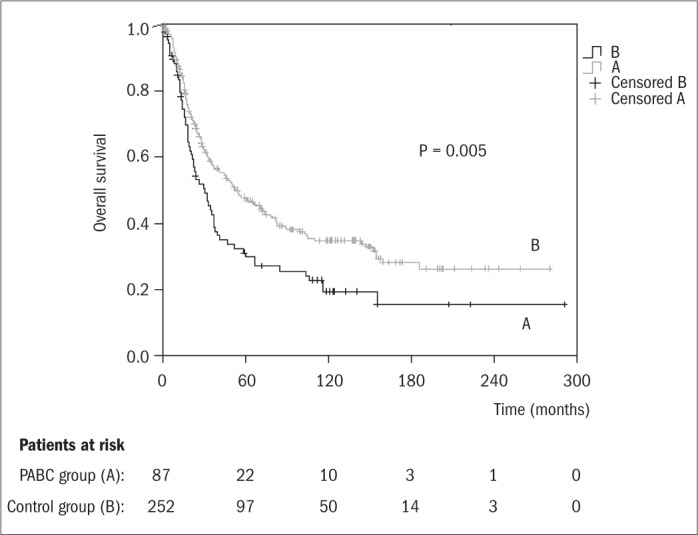
Kaplan-Meier overall survival functions for pregnancy-associated breast cancer (PABC) patients (trace A) and their corresponding controls (trace B). Median values: (A) 30.1 months (95% confidence interval, CI = 19.4-40.9); (B) 53.1 months (95% CI = 35.1-71.0).

As can be seen from Figure 1, the median overall survival of the PABC group was 30.1 months (95% confidence interval, CI = 19.4-40.9 months), while that of the control group was 53.1 months (95% CI = 35.1-71.0 months). Overall survival within a five-year period was 29.7% for the PABC group and 47.3% for the control group, while within a 10-year period, the overall survival rates were 19.2 and 34.8%, respectively. These results clearly demonstrate that the overall survival of patients with breast cancer was significantly lower (P = 0.005) when this condition was associated with pregnancy.

### Prognostic factor analysis

In order to investigate whether the difference demonstrated between the overall survival of patients was in fact a consequence of pregnancy and not influenced by concomitant confounding factors, prognostic factor analysis was performed. Univariate analysis revealed that the critical covariables influencing prognosis (P ≤ 0.20, as determined by the Mantel-Cox log rank method) were the tumor histology (P = 0.004), size of the primary tumor (P = 0.057), distant metastasis (P = 0.022), grade of malignancy (P = 0.005), hormone receptor status (P = 0.003) and interval between first symptoms and diagnosing of cancer (P = 0.009).

The contributions of the covariables and pregnancy to the patients’ overall survival was analyzed in accordance with the Cox regression method and the results are presented in Table 3. The factors independently associated with overall survival were the size of the primary tumor, distant metastasis, grade of malignancy and pregnancy. Thus, pregnancy significantly reduced the overall survival of young women (aged ≤ 45 years) with breast cancer, independent of other factors.

**Table 3.** Multivariate analysis on the covariables associated with overall survival of patients diagnosed with pregnancy-associated breast cancer (Cox multivariate model)

Covariables	Degrees of freedom	P*	Hazard ratio
Tumor histology	1	0.863	—
Tumor size	1	< 0.0001	1.62
Distant metastasis	1	< 0.0001	5.08
Grade of malignancy	2	< 0.0001	
1 and 2 versus 3	1	0.002	0.54
1 and 2 versus unknown	1	0.226	—
Hormone receptor status	2	0.162	—
Positive versus negative	1	0.945	—
Not determined versus negative	1	0.066	—
Pregnancy	1	0.011	1.52
Interval between first symptoms and diagnosis	1	0.207	—

* Values based on χ^2^ test (Wald method).

**Figure 2:**
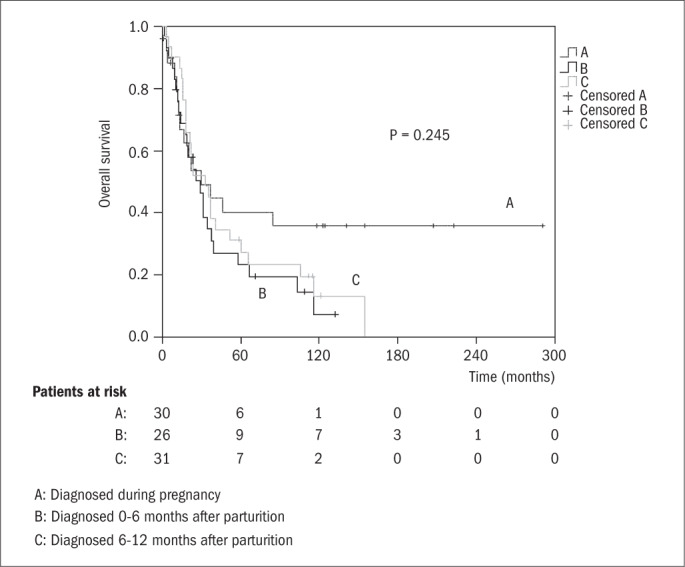
Kaplan-Meier overall survival functions for pregnancy-associated breast cancer (PABC) patients diagnosed during pregnancy (trace A), diagnosed 0-6 months after parturition/abortion (trace B) and diagnosed 6-12 months after parturition/abortion (trace C). Median values: (A) 29.17 months (95% confidence interval, CI = 14.47-43.86); (B) 30.13 months (95% CI = 4.69-55.57); (C) 32.63 months (95% CI = 14.52-50.74).

**Figure 3:**
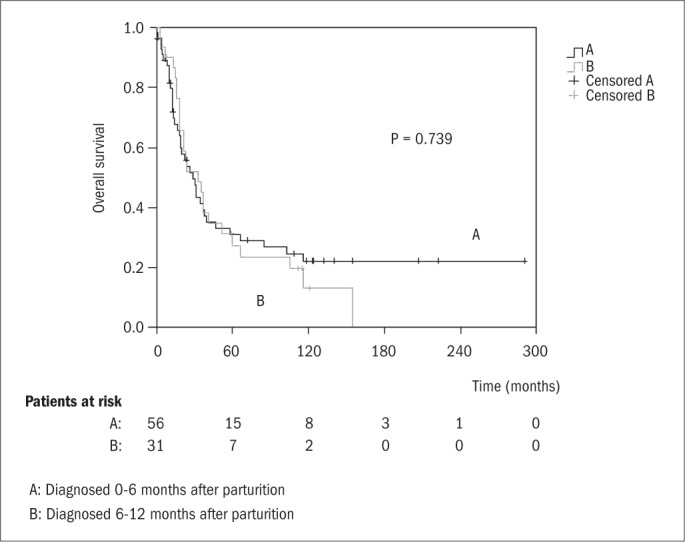
Kaplan-Meier overall survival functions for pregnancy-associated breast cancer (PABC) patients diagnosed during pregnancy or 0-6 months after parturition/abortion (trace A) and diagnosed 6-12 months after parturition/abortion (trace B). Median values: (A) 29.16 months (95% confidence interval, CI = 18.53-39.80); (B) 32.63 months (95% CI = 14.52-50.74).

In order to determine whether prognosis was associated with the time at which cancer was diagnosed, the overall survival functions were plotted in relation to three subgroups of PABC patients according to the following models: (i) diagnosis during pregnancy versus diagnosis 0-6 months after parturition versus diagnosis 6-12 months after parturition (Figure 2); and (ii) diagnosis during pregnancy or 0-6 months after parturition versus diagnosis 6-12 months after parturition (Figure 3). No significant differences were found between these two models, thus indicating that the date of diagnosing the cancer in relation to parturition did not significantly influence the prognosis.

## DISCUSSION

It became clear during the design phase of this project that multivariate analysis on prognostic factors in a paired-case control study would be a powerful tool through which to evaluate the interdependency of the different covariables. Hence, following published methodology,^6,8,11^ the PABC patients were matched with corresponding controls based on the institution of registration, year of registration and patient's age (± two years). The year of registration was used as the criterion for the purpose of obtaining uniformity regarding the type of diagnostic tests, classification systems and treatment. Indeed, the date of confirmation of diagnosis, as provided by the anatomopathological test, was close to the date of registration at the institution. Thus, it was a precise figure that could be obtained from the medical records.

The proportional hazard regression model proposed by Cox is often used when the outcome of an event corresponds to a time interval and when some data are censored. This method is considered valid and is in agreement with more complex methods of analysis, as long as the number of covariables is less than 10% of the number of events. Within the population of 339 women studied, the number of death events was 213 (62.8% of the initial population), while the number of covariables was 8. Hence, the level of agreement between the predictable and observable values should be 96.5%.^14^

In the present study, a pessimistic prognosis for the overall survival of PABC patients, compared with the control patients, was demonstrated. As early as 1943, Haagensen^15^ had reported that breast carcinoma that developed during pregnancy or during the lactating phase tended to be so malignant that surgery could not be recommended as part of the therapy. Although the study was based on the outcomes of just 20 patients, all of whom died as a consequence of neoplasia, and despite the author's subsequent reconsideration of his opinion, the concept of the inoperability of PABC patients prevailed for many years.^15^

Following a review of 700 PABC cases (some dating back to 1880), White^5^ concluded that the prognosis for such patients was worse than for their non-pregnant counterparts and that this was mainly attributable to delayed diagnosis and treatment. Petrek et al.^10^ used logistic regression analysis to investigate axillary lymph node involvement and pregnancy as possible predictive factors for the emergence of breast cancer in 56 PABC patients in various stages of the disease. Although these authors found that only axillary lymph node involvement was decisive for the prognosis of the outcome, they reported that delayed diagnosis during pregnancy or postpartum resulted in advanced breast cancer. Delayed diagnosis and treatment, together with a more aggressive condition, were considered by Ishida et al.^6^ to be responsible for the poor prognosis presented by 192 patients diagnosed with breast cancer during pregnancy or up to two years after parturition, in comparison with 191 non-pregnant and non-lactating breast cancer patients of approximately the same age. These authors found that the incidence of axillary lymph node involvement and negative hormone receptor status was higher amongst pregnant and lactating women than among controls, although the lack of multivariate analysis precluded determining the relative contribution of each of these factors. A recent study conducted by Rodriguez et al.,^9^ involving 797 PABC patients and 4,177 controls, concluded that pregnancy significantly (P = 0.046) worsened the prognosis for breast cancer, even when other factors were taken into consideration.

Most investigations concerned with the overall survival of PABC patients have concluded that the prognosis for such patients is more pessimistic than for non-pregnant patients. One simple explanation for this is that pregnancy is characterized by increased estrogen concentration in the body, which is a well-known breast cancer-promoting factor. Other explanations relate to causes that are not so obvious. A randomized prospective clinical experiment would clarify this matter, since the influence of unknown confounding factors would be normalized. However, since such an experiment would be impracticable, case-control studies are the best available evaluation method.

A number of case-control studies on PABC patients have been published over the last decade or so. Bonnier et al.^8^ studied 154 PABC patients who were diagnosed with cancer either during pregnancy or no more than six months after parturition, and 308 control patients with breast cancer that was not associated with pregnancy or the postpartum period, all of whom had been registered in 23 French institutions during the period 1960-1993. The PABC group was matched to the control group in the proportions of 1:2, according to age and date of commencing the treatment. Multivariate analysis (using the Cox regression method) revealed that tumor size (≤ 3.0 cm versus > 3.0 cm), axillary lymph node involvement (0 versus 1-3 versus ≥ 4) and age (≤ 33 years versus > 33 years) each exerted an independent and significant influence on overall survival and on the emergence of metastasis. In contrast, Ibrahim et al.^11^ investigated 72 PABC patients together with their matching controls (216 non-pregnant women in the same condition), who were selected according to disease stage, year of diagnosis and age. Only disease stage was identified as significant with regard to overall survival according to the Cox model.

In the present study, the independent influence of the covariables of interval between first symptoms and diagnosis, tumor histology, size of primary tumor, distant metastasis, grade of malignancy, hormone receptor status and axillary lymph node involvement and the influence of the pregnancy variable on the patients’ overall survival were investigated. Overall survival was independently associated with primary tumor size, grade of malignancy, distant metastasis and pregnancy. The interval between first symptoms and diagnosis and the interval between diagnosis and first treatment had no significant influence on the prognosis, and hence, the hypothesis that delayed diagnosis and treatment is a cause of pessimistic prognosis could not be confirmed.

Some authors have attributed the pessimistic prognosis for PABC women to the difficulties in diagnosing cancer in this group of patients.^5,10^ In fact, the diagnosis of breast cancer in patients younger than 40 years is more difficult because of the characteristics of mammary tissue at this age.^16^ Additionally, in spite of the dissimilar criteria used for patient selection and the somewhat conflicting results, a number of reports^6,8,11^ have suggested that the interval between cancer diagnosis and partum could be an important factor for prognosis and overall survival. Thus, on the basis of a retrospective study involving 407 women aged 20-29 years who were diagnosed with cancer during pregnancy or with a history of pregnancy (no limitation of time) prior to diagnosis, Guinee et al.^7^ proposed that the shorter the interval between pregnancy and the diagnosing of cancer was, the more negative the prognosis would be. Moreover, following a population-based study involving 4,299 women (20-54 years old) who had been diagnosed with breast cancer, Whiteman et al.^17^ also concluded that the shorter the interval between diagnosing the cancer and the last partum was, the worse the prognosis of the disease was.

However, the findings reported in the present paper do not support this hypothesis, as can be seen from Figures 2 and 3. In contrast to the earlier investigation, the present study included only patients whose pregnancy occurred up to a maximum of 12 months prior to diagnosing the cancer. Perhaps the difference between the results from these two groups was not large enough to be detected by the tests used. For this reason, no unambiguous conclusion can be drawn from the present results regarding this point.

## CONCLUSIONS

Since many of the studies conducted so far have reported divergent results, it is clear that the factors associated with the overall survival and prognosis for PABC patients require further investigation. However, it may be concluded from the present study that the overall survival of PABC patients is significantly lower than for other patients suffering from breast cancer, and that pregnancy is a factor associated with a pessimistic prognosis, independent of other concomitant factors.
